# Antidepressant-Like Effects of *Lindera obtusiloba* Extracts on the Immobility Behavior of Rats in the Forced Swim Test

**DOI:** 10.3390/molecules21030277

**Published:** 2016-02-27

**Authors:** Dong Wook Lim, Mi-Sook Lee, Song Her, Suengmok Cho, Chang-Ho Lee, In-Ho Kim, Daeseok Han

**Affiliations:** 1 Research Group of Innovative Special Food, Korea Food Research Institute, Seongnam 463-746, Korea; neodw4015@kfri.re.kr (D.W.L.); smcho@kfri.re.kr (S.C.); chang@kfri.re.k (C.H.L.); skihs@kfri.re.kr (I.H.K.); 2 Division of Bio-Imaging, Chuncheon Center, Korea Basic Science Institute, Chuncheon 200-701, Korea; mslee0621@gmail.com (M.S.L.); swher@kbsi.re.kr (S.H.)

**Keywords:** *Lindera obtusiloba*, depression, hypothalamic-pituitary-adrenal axis, glucocorticoid receptor, forced swim test

## Abstract

*Lindera obtusiloba* extracts are commonly used as an alternative medicine due to its numerous health benefits in Korea. However, the antidepressant-like effects of *L. obtusiloba* extracts have not been fully elucidated. In this study, we aimed to determine whether *L. obtusiloba* extracts exhibited antidepressant-like activity in rats subjected to forced swim test (FST)-induced depression. Acute treatment of rats with *L. obtusiloba* extracts (200 mg/kg, p.o.) significantly reduced immobility time and increased swimming time without any significant change in climbing. Rats treated with *L. obtusiloba* extracts also exhibited a decrease in the limbic hypothalamic-pituitary-adrenal (HPA) axis response to the FST, as indicated by attenuation of the corticosterone response and decreased c-Fos immunoreactivity in the hippocampus CA3 region. In addition, *L. obtusiloba* extracts, at concentrations that were not affected by cell viability, significantly decreased luciferase activity in response to cortisol in a concentration-dependent manner by the glucocorticoid binding assay in HeLa cells. Our findings suggested that the antidepressant-like effects of *L. obtusiloba* extracts were likely mediated via the glucocorticoid receptor (GR). Further studies are needed to evaluate the potential of *L. obtusiloba* extracts as an alternative therapeutic approach for the treatment of depression.

## 1. Introduction

Continued and elevated glucocorticoid levels resulting from dysfunction of the hypothalamic-pituitary-adrenal (HPA) axis is one of the most prominent neurobiological findings in depression [1,2]. The glucocorticoid receptor (GR) mediates the direct effects of the glucocorticoids that are released in response to stress and regulates the HPA axis via a negative feedback mechanism in the hippocampus, hypothalamus, and pituitary gland [3,4]. Previous clinical studies have reported that depressed subjects exhibit down-regulation of GR expression, which subsequently leads to an increase in the endogenous levels of glucocorticoids [5]. Thus, GR function may be one of the potential mechanisms underlying HPA axis dysfunction [6]. Consistent with a role of glucocorticoids in depression, GR antagonists have been encouraged for potential therapeutic benefits in stress-related disorders. This is based on the ability of GR antagonists to block the increase in the levels of circulating glucocorticoids and on their ability to up-regulate GR [7].

Recently, many herbal extracts have been shown to have antidepressant-like effects in a variety of animal models. For example, *Hypericum perforatum*, also known as St. John’s wort, is widely used for the treatment of mild to moderate depression [8]. *Panax ginseng*, commonly known as Korea Ginseng, has been investigated experimentally and clinically for its stress-attenuating activity [9,10]. *Lindera obtusiloba* BLUME (Lauraceae), a ubiquitous tree distributed mainly in Southeast Asia has been used in traditional Chinese medicine [11]. Especially, *L. obtusiloba* leaf or branch extracts are a remedy that is widely used in Korean traditional medicine for the treatment of liver disease, insomnia, anxiety and for improving blood circulation [12]. *L. obtusiloba* has also been reported to possess anti-inflammatory [13], neuroprotective [14], anti-fibrotic [15], and anti-hepatotoxic [16] effects. However, little is known about the antidepressant-like effects of *L. obtusiloba* extracts, and their efficacy needs to be scientifically evaluated in *in vivo* experiments.

In the present study, the antidepressant-like effects of *L. obtusiloba* extracts were investigated in response to the forced swim test (FST) in rats. Moreover, to determine the neurobiological effects underlying the antidepressant-like activity of the *L. obtusiloba* extracts, corticosterone responses and c-Fos immunoreactivity were evaluated in rats exposed to FST. Finally, we also examined the antiglucocorticoid activity of *L. obtusiloba* extracts using the glucocorticoid binding assay in HeLa cells.

## 2. Results

### 2.1. Effect of L. obtusiloba Extracts on Depressant Behaviors in Response to the FST

**Figure 1:**
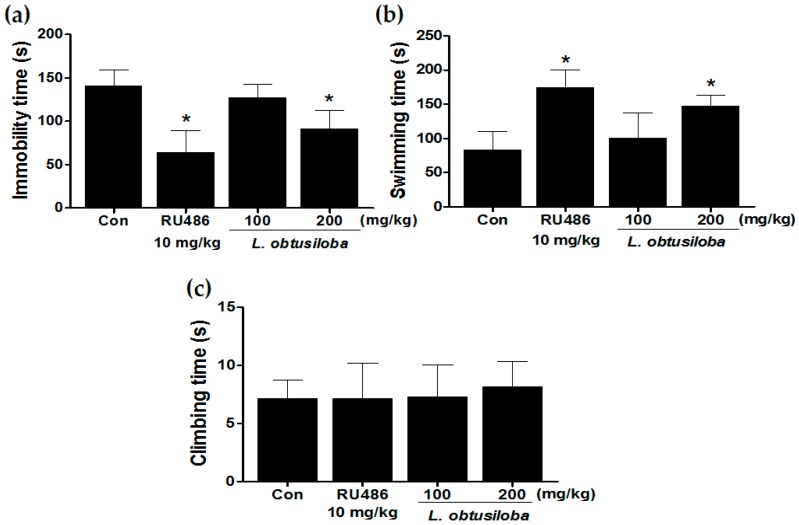
Antidepressant-like effects of treatment with *L. obtusiloba* extracts on depressive behavior in response to the FST. Immobility (**a**); swimming (**b**); and climbing (**c**) were recorded during the last 4 min in the FST. Columns show the means ± SEMs (*n* = 10). * *p* < 0.05 *vs.* the control group.

We examined the antidepressant-like effects of *L. obtusiloba* extracts in the FST. *L. obtusiloba* extract treatment in rats reduced the duration of immobility, reducing immobility by a maximum of 35.67% when administered at a dose of 200 mg/kg (Figure 1A). *L. obtusiloba* extracts also significantly increased the swimming time without any significant change in climbing (Figure 1B). RU 486 (10 mg/kg), which was used as the positive control, markedly decreased immobility time and increased swimming time in the FST.

### 2.2. Effect of L. obtusiloba Extracts on Serum Corticosterone Levels

**Figure 2:**
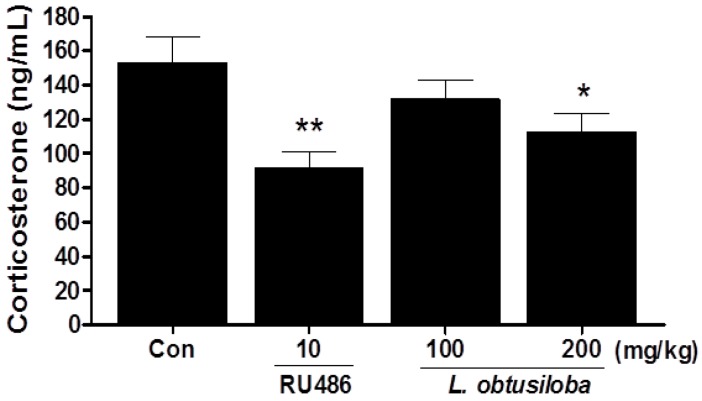
Effects of *L. obtusiloba* extracts on serum corticosterone levels. Blood samples were collected via the abdominal aorta after the FST for corticosterone analysis. Columns show the means ± SEMs (*n* = 10). * *p* < 0.05 and ** *p* < 0.01 *vs.* the control group.

Serum corticosterone levels were significantly decreased in rats treated with 200 mg/kg *L. obtusiloba* extracts compared with that in the control group (Figure 2). Similar results were observed in RU 486-treated rats.

### 2.3. Effect of L. obtusiloba Extracts on c-Fos Expression in the Hippocampus CA3 Region

**Figure 3:**
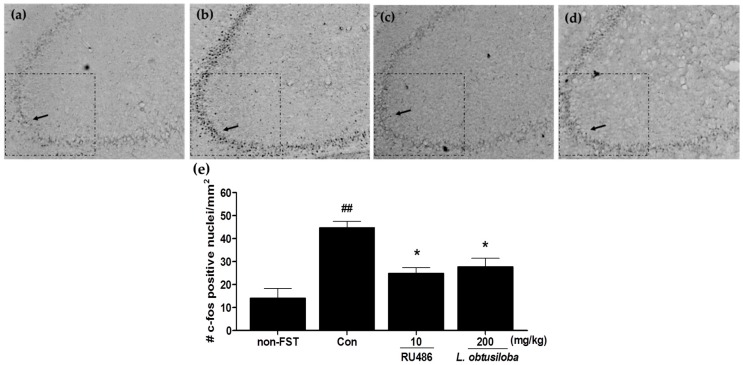
Effects of *L. obtusiloba* extracts on c-Fos expression in the CA3 region of the hippocampus. Representative photomicrographs show c-Fos–positive nuclei in the CA3 of normal (non-exposed to FST) (**a**); control (**b**); RU 486 (10 mg/kg)-treated (**c**); and *L. obtusiloba* extracts (200 mg/kg)-treated (**d**) rats exposed to the FST. Arrows indicate examples of c-Fos positive neurons. Columns show the means ± SEMs (*n* = 5) values of c-Fos expression in the CA3 region of the hippocampus (**e**). These data were evaluated by Kruskal-Wallis median test (*p* < 0.01) and validated by the Dunn’s multiple comparison test (*p* < 0.05). * *p* < 0.05 *vs.* the control group. ^##^ *p* < 0.01 *vs.* the non-exposed to FST.

To examine whether *L. obtusiloba* extracts affected the neural responses in rats exposed to FST, c-Fos expression was measured in the hippocampus using immunohistochemistry. Increased activation of c-Fos was observed in the CA3 region of the hippocampus following the FST in vehicle-treated rats. c-Fos expression in RU 486–treated rats was similar to that previously reported for stress-induced c-Fos expression in the CA3 region of the hippocampus [17]. Importantly, treatment with 200 mg/kg *L. obtusiloba* extracts significantly inhibited c-Fos activity as compared with that in the vehicle-treated control group (Figure 3).

### 2.4. Effect of Antagonistic Activity of RU 486 and L. obtusiloba Extracts on Cortisol-Induced GR Transactivation

**Figure 4:**
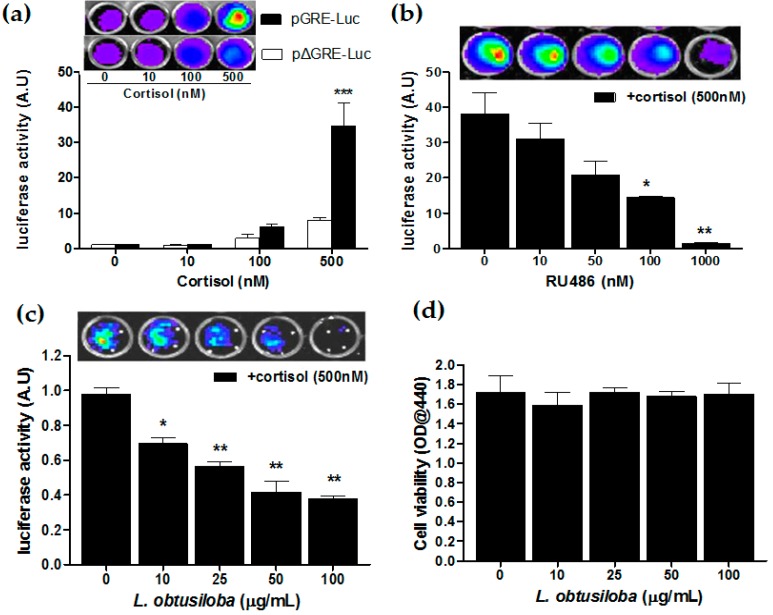
Inhibition of GR transactivation by *L. obtusiloba* extracts. Cortisol-induced GR transactivation in HeLa cells (**a**). After HeLa cells were transfected with the pGRE-Luc 12 h before cortisol treatment. Construct-deleted 5xGRE DNA fragment (pΔGRE-Luc) was used as a negative control. *** *p* < 0.001 *vs.* pΔGRE-Luc. Inhibition of GR transactivation by RU 486 (**b**). pGRE-Luc was transfected into HeLa cells, and the transfected cells were treated with the indicated doses of RU 486 3 h before stimulation with 500 nM cortisol. * *p* < 0.05 and ** *p* < 0.01 *vs.* non-treatment (+cortisol). Inhibition of GR transactivation by *L. obtusiloba* extracts (**c**). pGRE-Luc was transfected with the pGRE-Luc into HeLa cells, and the transfected cells were treated with the indicated doses of *L. obtusiloba* extracts 3 h before stimulation with 500 nM cortisol. * *p* < 0.05 and ** *p* < 0.01 *vs.* non-treatment (+cortisol). Results are normalized for transfection efficiency and plotted as arbitrary units (A.U) in triplicate samples from three independent transfections. Effect of *L. obtusiloba* extracts on HeLa cell viability (**d**). Viability of cells was assessed by WST-1 assay. All results are expressed as mean ± SEMs.

We have carried out the GR transactivation in the presence of glucocorticoids using the luciferase reporter assay. HeLa cells transfected with pGRE-Luc were treated with cortisol, at an indicated concentration. Then, 36 h after cortisol treatment, the cells increased luciferase reporter activity in a concentration-dependent manner with a statistical significance in cortisol treatment of 500 nM compared with non-treatment. At a concentration of 500 nM, transfected cells with pGRE-Luc exhibited a 4.1-fold greater GR transactivation compared to pΔGRE-Luc–transfected cells (*** *p* < 0.001; Figure 4a). Then, HeLa cells harboring the GRE-Luc construct were treated with a GR antagonist, RU 486, 3 h before 500 nM corticosterone treatment. The RU 486 strongly blocked cortisol-activated GR transactivation in a concentration-dependent manner. At a concentration of 1000 nM, RU 486 showed a 21.52-fold greater transrepressive effect compared with non-treatment (** *p* < 0.01; Figure 4b). Importantly, the addition of *L. obtusiloba* extracts decreased in cortisol-activated GR transactivation in a concentration-dependent manner. At a concentration of 100 µg/mL, *L. obtusiloba* extracts significantly decreased in luciferase activity, showing a 2.73-fold transrepressive effect (** *p* < 0.01; Figure 4c). The inhibition of cortisol-mediated GR transactivation by *L. obtusiloba* extract treatment did not appear to be influenced by inhibition of cell viability (Figure 4d).

## 3. Discussion

In the present study, we examined the antidepressant-like effects of *L. obtusiloba* extracts in FST-induced depression in rats. Our results demonstrated that acute treatment with *L. obtusiloba* extracts significantly decreased the immobility time in rats exposed to the FST. Moreover, *L. obtusiloba* extracts decreased the hypothalamic-pituitary-adrenal (HPA) axis response to stress, as indicated by attenuation of the corticosterone response and decreased c-Fos immunoreactivity in the CA3 region of hippocampus.

Animal models of depression play an important role in the screening and evaluation of antidepressants [18]. The FST is an effective screening tool with good reliability and predictive validity [19]; the state of immobility in the FST is reported to mimic the symptoms of depression in humans and can be reversed by treatment with antidepressant drugs [20]. In our study, treatment with *L. obtusiloba* extracts (200 mg/kg) decreased immobility and increased swimming time without any significant change in climbing. This pattern has also been observed by treatment with paroxetine and fluoxetine, selective serotonin reuptake inhibitors (SSRIs) [21,22]. In this regard, many natural compounds have been shown to influence FST and serotonin levels [23]. The antidepressant-like effects of *L. obtusiloba* extracts were also investigated by quantitative analysis of c-Fos immunoreactivity and analysis of the activity of the HPA axis, which are both associated with high corticosterone production [24]. The c-Fos is an immediate-early gene that is rapidly expressed in response to neuronal activation [25] and has been widely used as a marker for neuronal activation and to explore the effects of external stimuli on neuronal circuits [26]. Previous studies involving acute or chronic stress states have demonstrated that profound alterations in the expression of glucocorticoid receptor (GR) mRNA are closely associated with elevated corticosterone production and c-Fos expression [27]. Antidepressant drugs, including SSRIs, compensate for impaired feedback inhibition by regulating GR levels in the hippocampus [28]. We found that treatment with *L. obtusiloba* extracts at a dose of 200 mg/kg inhibited the increase in c-Fos–positive cells in the hippocampus CA3 region associated with stress-induced depression, and reduced the HPA axis response to stress, as indicated by the decrease in serum corticosterone levels. These results suggested that *L. obtusiloba* extracts exhibited significant antidepressant-like effects in the FST.

The RU 486, Mifepristone, as a positive control, possesses potent antagonistic activity against hormones (progesterone and glucocorticosteroids) [29]. Numerous studies have demonstrated a significant reduction in depressive symptoms in patients treated with RU 486 [5,30,31]. In our results, treatment with RU 486 (10 mg/kg) decreased immobility times in the FST, and recently it has been reported that GR antagonists reversed the increased immobility time in the FST [32,33]. In addition, RU 486 normalizes the chronic stress– and corticosterone-induced reduction of hippocampal neurogenesis [34], which may contribute to the hippocampal volume reductions observed in depression [35]. These reports support the notion that GR antagonists may be useful as antidepressants for the treatment of depression, via the regulation of the HPA axis [36]. Consistent with this idea, we also investigated the anti-glucocorticoid activity of *L. obtusiloba* extracts *in vitro*, using the hormone responsive element reporter assay. *L. obtusiloba* extracts, at concentrations that were not affected by cell viability, significantly decreased luciferase activity in response to cortisol in a concentration-dependent manner. These results strongly suggested that the antidepressant-like effects of *L. obtusiloba* extracts were likely mediated via GR. However, further studies are needed to determine the GR phosphorylation and GR-dependent transactivation in the hippocampus.

## 4. Materials and Methods

### 4.1. Preparation of L. obtusiloba Extracts

Dried powdered *L. obtusiloba* (500 g, Kapdang, Seoul, Korea) was prepared after immersion in ethanol (5 L) using an ultrasonic cleaning bath (model 5510, Branson, Danbury, CT, USA) for 48 h. The process was repeated once, and the extracts were combined and filtered through a membrane filter (0.45 µm; Millipore, Billerica, MA, USA). After removing the solvents via rotary evaporation, the remaining extracts were vacuum dried to a yield of about 5.2% (*w*/*w*). The dry extract was then dissolved in distilled water.

### 4.2. Animals

Male Sprague Dawley (SD) rats (eight weeks old; Samtako Bio Korea, Gyeonggi-do, Korea) weighing 180–210 g were housed at two rats per cage under a controlled temperature (23 ± 1 °C) and a 12-h light/dark cycle (lights on at 07:00 and lights off at 19:00). The rats were allowed at least one week for acclimatization before the experiments. All animal protocols were approved by the Institutional Animal Care and Use Committee (IACUC) of Korea Food Research Institute.

### 4.3. Treatments

RU 486 (Sigma, St. Louis, MO, USA) dissolved in 80% sesame oil was used as a positive control of antidepressant activity. Two doses of *L. obtusiloba* extracts (100 and 200 mg/kg, p.o., *n* = 10 per group) or a dose of RU 486 (10 mg/kg, i.p., an injection volume of 0.1 mL/100 g body weigh) were treated for seven consecutive days. The control group was administered the vehicle solution (1 mL/kg, p.o.) using the same schedule of administration. On day 7, 1 h after the administration, the rats were exposed to the behavioral experiments.

### 4.4. Forced Swim Test

The FST was carried out as previously described [37]. Briefly, in the pretest session, rats were forced to swim for 15 min in a transparent Plexiglas cylinder (height 50 cm; diameter 20 cm) filled to a depth of 30 cm with water (temperature, 23–25 °C). Twenty-four hours later, the procedure was repeated during a 6-min test session, and the immobility time during the last 4 min was measured by a SMART video tracking system (SMART v3.0, Panlab SL, Barcelona, Spain). Rats were considered immobile when they ceased struggling, remained floating motionless, and only made movements necessary to keep their heads above the water [38]. Behavior data were analyzed by trained experimenters blind to the treatment groups.

### 4.5. Serum Corticosterone Assay

Blood samples were collected via the abdominal aorta after the FST. The serum samples were prepared by centrifugation of the collected blood samples for 15 min at 13,000× *g* within 30 min and stored frozen (−80 °C). The serum levels of corticosterone were measured using commercially available enzyme immunoassay kits (DetectX; Arbor Assays, Ann Arbor, MI, USA).

### 4.6. Immunohistochemistry

Rats were sacrificed following the FST test, and their brains were fixed through the ascending aorta with 0.9% saline, followed by 500 mL of cold 0.1 M phosphate buffer (PB) containing 4% paraformaldehyde (PFA). The fixed brains were cut into 30 µm sections on a cryostat (CM1850; Leica, Heidelberg, Germany). Immunohistochemistry staining was performed on 30-µm sections using polyclonal antibodies specific for c-Fos (1:500 dilution; Santa Cruz Biotechnology, Santa Cruz, CA, USA), followed by exposure to a biotinylated anti-rabbit antibody (1:500 dilution; cat. no. BA1000; Vector Labs, Burlington, ON, Canada). The sections were reacted with an avidin-biotin-peroxidase complex (Elite ABC kit; 1:50 dilution; Vector Labs) at room temperature for 60 min, and the avidin-biotin complex was visualized with 0.05% 3,3-diaminobenzidine (DAB; Sigma) and 0.02% H_2_O_2_. Images of immunohistochemically stained sections were captured by a camera mounted on an Olympus BX-51 microscope (Olympus Optical, Tokyo, Japan). The number of c-Fos-positive cell nuclei within each area was counted bilaterally (where possible) in four to six consecutive sections per animal in a blind fashion, and the average of them was expressed as number of cells.

### 4.7. Cell Culture

HeLa cells were obtained from the American Type Culture Collection (ATCC; Manassas, VA, USA) and grown in Dulbecco’s modified Eagle’s medium (DMEM) supplemented with 10% fetal bovine serum (FBS), 100 U/mL penicillin, and 100 U/mL streptomycin under 5% CO_2_ at 37 °C, as recommended by the supplier.

### 4.8. Luciferase Enzyme Assays

HeLa cells were cotransfected with pGRE-Luc harboring a luciferase reporter gene [39]. As a negative control, the same construct with a deleted GRE DNA fragment (pΔGRE-Luc) was used. Transfection was performed using Effectene transfection reagent (QIAGEN, Valencia, CA, USA) according to the instruction manual. The transfected cells were incubated for 36 h with cortisol (Sigma), RU486 (Sigma), or *L. obtusiloba* at the specified concentration mentioned in the legends of the figures. Luciferase activity was measured using an IVIS 200 system (Caliper, Hopkinton, MA, USA), as described previously [40]. The reporter activity was analyzed by Living Image 2.60 software (Caliper), and then normalized for transfection efficiency with total protein (BCA Protein Assay-RAC; Pierce, Rockford, IL, USA). Results were expressed as fold activity per mg cellular protein.

### 4.9. Cell Viability Assay

The HeLa cells were prepared in 96-well plates with 2 × 104 cells per well. After 24 h, the medium was switched to the DMEM phenol red-free medium containing the *L. obtusiloba* extracts and was assayed with a WST-1 colorimetric assay kit (Takara, Kyoto, Japan) at 36 h.

### 4.10. Statistical Analysis

*In vivo* data analysis was performed using one-way analysis of variance (ANOVA) followed by Tukey’s *post-hoc* test using Prism 5 (GraphPad Software, Inc., San Diego, CA, USA) for multigroup comparisons. Non-normally distributed data of c-Fos expression was analyzed by Kruskal-Wallis median test combined with Dunn’s multiple comparison test. For *in vitro* data analysis was performed using two-way ANOVA with Bonferroni *post hoc* test or Student’s *t*-test. Results of *p* < 0.05 were considered statistically significant. All results are expressed as mean ± standard error of the mean (SEMs).

## 5. Conclusions

In conclusion, our results demonstrated that treatment with *L. obtusiloba* extracts significantly decreased immobility time and the HPA axis response in rats exposed to the FST, as indicated by attenuation of the corticosterone response and decreased c-Fos immunoreactivity in the hippocampus CA3 region. In addition, this was the first study to show that *L. obtusiloba* extracts cause antidepressant-like effects through interaction with the GR. Thus, our findings suggest that *L. obtusiloba* extracts may offer possible advantages over herbal remedies for depressive disorder.

## Abbreviations

The following abbreviations are used in this manuscript:

HPA: Hypothalamic-pituitary-adrenal
GR: Glucocorticoid receptor
FST: Forced swim test
PB: Phosphate buffer
DAB: 3,3-diaminobenzidine
DMEM: Dulbecco’s modified Eagle’s medium
FBS: Fetal bovine serum
pGRE-Luc: Androgen and glucocorticoid responsive firefly luciferase reporter vector
pΔGRE-Luc: A deleted GRE DNA fragment
